# Low-Pass Parabolic FFT Filter for Airborne and Satellite Lidar Signal Processing

**DOI:** 10.3390/s151026085

**Published:** 2015-10-14

**Authors:** Zhongke Jiao, Bo Liu, Enhai Liu, Yongjian Yue

**Affiliations:** 1 Institute of Optics and Electronics, Chinese Academy of Sciences, Mailbox 350, Chengdu 610209, China; E-Mails: jiaozhongke2008@163.com (Z.J.); leh@ioe.ac.cn (E.L.); yankunioe@163.com (Y.Y.); 2 Department of Atmospheric Science, University of Wyoming, Laramie, WY 82071, USA

**Keywords:** (010.3640) lidar, (010.1615) clouds, signal, noise

## Abstract

In order to reduce random errors of the lidar signal inversion, a low-pass parabolic fast Fourier transform filter (PFFTF) was introduced for noise elimination. A compact airborne Raman lidar system was studied, which applied PFFTF to process lidar signals. Mathematics and simulations of PFFTF along with low pass filters, sliding mean filter (SMF), median filter (MF), empirical mode decomposition (EMD) and wavelet transform (WT) were studied, and the practical engineering value of PFFTF for lidar signal processing has been verified. The method has been tested on real lidar signal from Wyoming Cloud Lidar (WCL). Results show that PFFTF has advantages over the other methods. It keeps the high frequency components well and reduces much of the random noise simultaneously for lidar signal processing.

## 1. Introduction

Lidar is an active remote sensing instrument, which measures the backscattering signals by emitting laser pulses towards atmosphere or targets. It is widely used in atmospheric remote sensing, such as detection of atmospheric aerosols, clouds, atmospheric boundary layer, temperature, visibility, and wind [1,2,3,4]. All of these measurements are based on the raw signal data processing. To improve the signal to noise ratio (SNR), except for the efforts on hardware, the signal processing is also crucial. The lidar return signal contains not only the backscattered signal produced by the atmosphere or targets, but also noises and interferences, such as stochastic and atmospheric turbulences, dark current, background and electronics readout noise [2,3,4,5]. The signal SNR falls rapidly with the increase of the detection distance, which results in that signals are often overwhelmed by noise. To reduce random noises, long-time averaging is an effective method for ground-based lidar systems. However, this is not an option for airborne and space borne systems due to the requirement of higher horizontal resolution. In this case, to enhance the distance and accuracy of the lidar detection, signal processing plays an indispensable role.

Other than traditional signal data smoothing, manifold signal processing algorithms and denoising methodologies were studied to process signals in various applications. Huang *et al.* put forward empirical mode decomposition (EMD) which is used to deal with the non-linear and non-stationary data [6]. It was reported that EMD method could also be applied to reduce noise in return lidar signals. Wu *et al.* proved its applicability, efficiency and superiority to the band-pass filter and the averaging method [2]. Tian *et al.* suggested an automatic EMD denoising method and proved the performance of the method [4]. Wavelet transform (WT) is another modern method studied by many researchers in processing signals. Fang *et al.* proposed discrete wavelet transform and improved the SNR and effective range of lidar [7]. Kedzierski and Fryskowska applied a wavelet-based method to process and integrate the return signal to generate the 3D model in lidar system [8]. EMD and WT might beat down noise in return lidar signal to some extent, while both of them are hardware resource-costing and time-consuming. Therefore, a method which is efficient and easily to be realized in engineering applications is also required, especially for real-time detection systems. In this paper, a low-pass parabolic fast Fourier transform filter (PFFTF) was introduced, which can be operated well for lidar signal denoising in real-time monitoring.

Theory and the procedure of PFFTF is illustrated in Section 2, and processing of a simulated lidar signal by PFFTF was presented and compared with traditional low pass filter (TLPF), triangular filter, Gaussian filter, sliding mean filter (SMF), median filter (MF), empirical mode decomposition (EMD) and wavelet transform (WT). The system design and setup of a compact airborne Raman lidar are introduced in Section 3. The performance of PFFTF for processing real lidar signals was tested and verified in Section 4. Finally, Section 5 concludes this paper.

## 2. Theory and Simulations

### 2.1. Theory and Procedure of PFFTF

**Figure 1:**
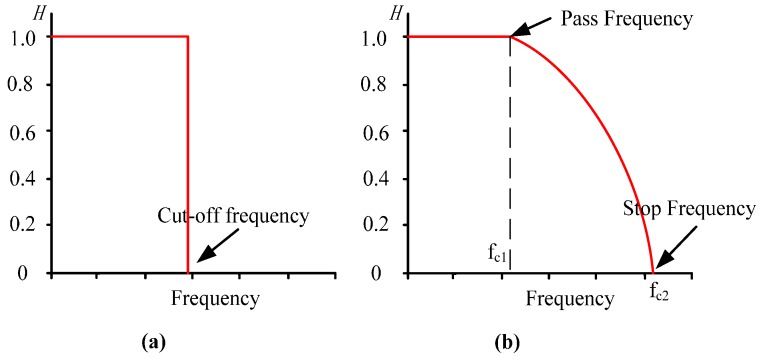
Window form of filters. (**a**) TLPF; (**b**) PFFTF.

For lidar signals, random noise is a high frequency component [4,6,9]. However, signals scattered by aerosol layers or clouds also have high frequency components [10,11,12]. For processing the signals, it is a challenge to reduce the random noise of the signal while keeping as many of the real high frequency signal details as possible. Fast Fourier transform (FFT) filter is a powerful tool to deal with the signal in the frequency domain. The frequency of the input signal is analyzed by FFT and then the required frequency components are selected. As shown in Figure 1a, traditional low-pass FFT filter blocks high frequency components completely. Different from it, the cut-off frequency of PFFTF is not ONE value, but a parabolic curve. Benefited from this change, the PFFTF retains partial high frequency components which are caused by clouds or aerosols.

The window function of PFFTF is expressed as Equation (1) [10], where f_C1_ is the pass frequency and f_C2_ is the stop frequency:

(1) $$H(f)=\{\begin{array}{lll} 1 & if & f\leq f_{C1}\\ 1-\frac{{\left(f-f_{C1}\right)}^{2}}{{\left(f_{C1}-f_{C2}\right)}^{2}} & if & f_{C1}\leq f\leq f_{C2}\\ 0 & if & f\geq f_{C2} \end{array}$$

**Figure 2:**
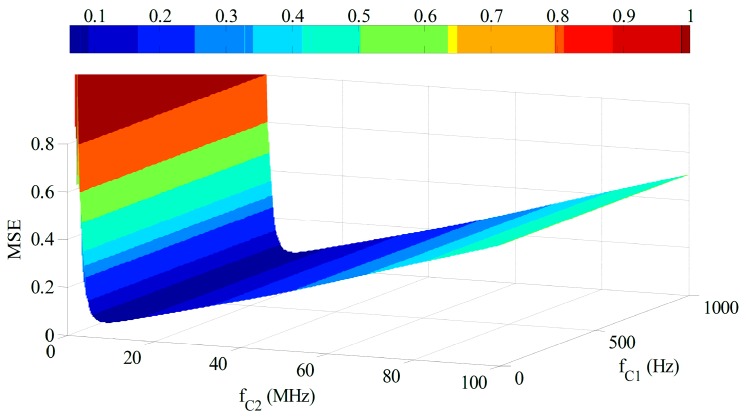
Variation of the MSE with f_C1_ and f_C2_.

The effect of PFFTF is determined by both the pass frequency and the stop frequency. Consequently, how to find out the optimal values for f_C1_ and f_C2_ is particularly significant. So far, no one has ever done research about how to select the pass and stop frequency of PFFTF. To evaluate the effect of different pass and stop frequencies when using PFFTF to process the return lidar signal, we choose mean square error (MSE) as a criterion, which is expressed in Equation (2). MSE reflects the deviation of processed signal from original signal. The MSE variations with f_C1_ and f_C2_ are shown in Figure 2, where the sampling frequency (f_S_) is 200 MHz:

(2) $$E_{MSE\_output}=\frac{1}{N}\sum_{n=1}^{N}{\left[x_{processed}(n)-x_{ideal}(n)\right]}^{2}$$

**Figure 3:**
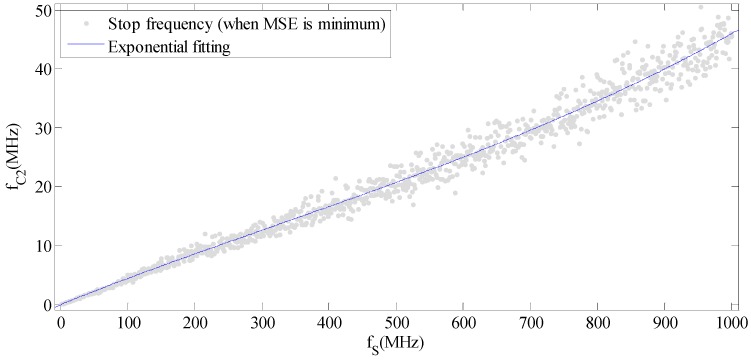
Relationship between f_S_ and f_C1_.

It can be seen that MSE is affected slightly by f_C1_, while it changes considerably with f_C2_. The optimal stop frequency can be computed when MSE is lowest. For lidar signals, the sampling frequency is usually between 1 MHz to 1 GHz. We compute the optimal values of stop frequency when the sampling frequency varies from 1 MHz to 1 GHz, as shown in Figure 3.

Experienced formulas for optimal stop frequency under different sampling frequency were obtained by exponential fitting, as shown in Equation (3):

(3) $$\left\{ \begin{array}{ll} f_{C1}=10 & 1\;\text{MHz}\leq f_{S}\leq 1000\;\text{MHz}\\ f_{C2}=13.42\times e^{0.001264\times {\text{f}}_{s}}-13.49\times e^{-0.002163\times {\text{f}}_{s}} & 1\;\text{MHz}\leq f_{S}\leq 1000\;\text{MHz} \end{array}\right.$$

**Figure 4:**
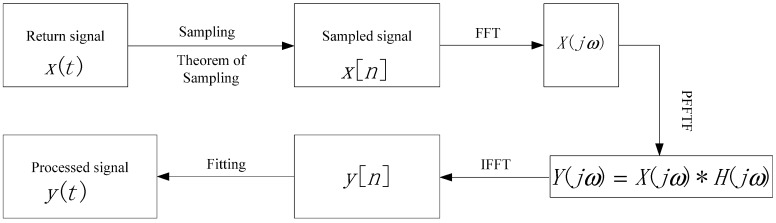
Procedure of PFFTF.

PFFTF is a kind of low pass filter, the essence of which is based on FFT and inverse fast Fourier transform (IFFT). The procedure of PFFTF is shown as Figure 4:

### 2.2. Simulations

To check the effects of the PFFTF, we did calculations for simulated lidar signals which were generated by lidar equations [1,13,14]. To make the simulation more real, we added Gaussian white noise and two fluctuation signals to the smooth ideal lidar signal. The simulated lidar signal is processed by TLPF, SMF, MF, triangular filter, Gaussian filter, WT, EMD and PFFTF.

SMF is based on the average method, which is commonly used for lidar signal processing. It smoothed both noise and signal, and lost some of the details. The application of SMF is defined by Equation (4):

(4) $$y(i)=\frac{1}{N}\sum_{j=-m}^{m}x(i+j)$$

where N=2m+1 and i=m+1,m+2,…,n−m.

MF is a method to process nonlinear signals, which uses the median instead of the average for the series. MF retains some of the fluctuation signals, but its effect for noise filtering is not satisfactory. The definition of MF is expressed by Equation (5)

(5) $$y_{i}=\underset{2p+1}{Med}\{x_{i-p},x_{i-p+1},\cdots ,x_{i-1},x_{i},x_{i+1},\cdots ,x_{i+p-1},x_{i+p}\}$$

where p=1,2,3,⋯ and i=p+1,p+2,p+3,⋯.

**Figure 5:**
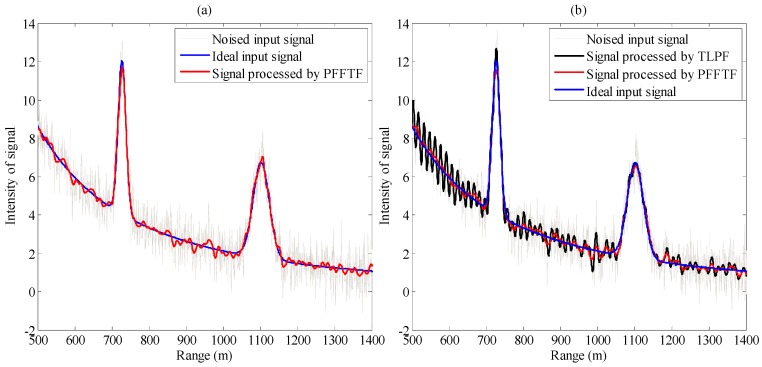

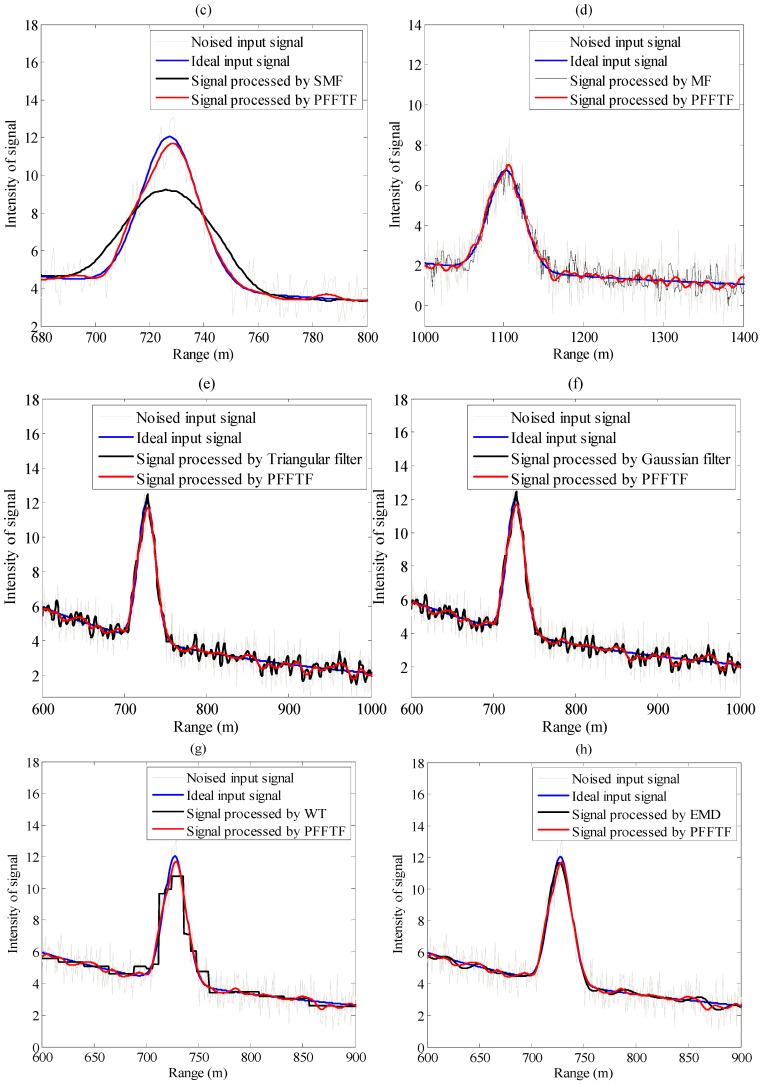
Comparison of different methods. (**a**) Comparison among ideal signal, noised input signal and signal processed by PFFTF; (**b**) Comparison between TLPF and PFFTF; (**c**) Comparison between SMF and PFFTF; (**d**) Comparison between MF and PFFTF; (**e**) Comparison between triangular filter and PFFTF; (**f**) Comparison between Gaussian filter and PFFTF; (**g**) Comparison between WT and PFFTF; (**h**) Comparison between EMD and PFFTF.

The Gaussian filter and triangular filter are time domain filters, which mathematically modify the input signal by convolution with an impulse response. They provide a smoother form of a signal, removing the short-term fluctuations, and leaving the longer-term trend. EMD is a method to decompose a signal into intrinsic mode functions (IMF) along with a trend and then remove proper IMF to get a processed signal [2,4,6]. WT is a transform that can decompose a signal into a set of basic functions and is applied to analyze signals [7,8]. The result of different methods is shown in Figure 5. In Figure 5, f_S_ is 200 MHz, f_C1_ is 10 Hz, f_C2_ is 8.86 MHz; the cut-off frequency of TLPF, triangular and Gaussian filter is 8.86 MHz, filter order of the triangular and Gaussian filter is 16; m is 15 for SMF, p is 2 for MF; for EMD, the interpolation method is “spline”, threshold of stop is 0.05; for WT, the wavelet is “haar” and the level is 6. PFFTF reduces the noise effectively and keeps the fluctuation signal well. It turns out that the empirical formulas of f_C1_ and f_C2_ are reasonable. In order to evaluate the denoising effects of these methods, SNR and MSE for input signal and output signal by different methods were computed respectively, which are expressed in Equations (6)–(9).

(6) $$R_{SNR\_input}=10\mathrm{lg}\frac{\sum_{k=1}^{K}f^{2}(k)}{\sum_{k=1}^{K}{\left[f_{n}(k)-f(k)\right]}^{2}}$$

(7) $$R_{SNR\_output}=10\mathrm{lg}\frac{\sum_{k=1}^{K}f^{2}(k)}{\sum_{k=1}^{K}{\left[f\prime (k)-f(k)\right]}^{2}}$$

(8) $$E_{MSE\_input}=\frac{1}{K}\sum_{k=1}^{K}{\left[f_{n}(k)-f(k)\right]}^{2}$$

(9) $$E_{MSE\_output}=\frac{1}{K}\sum_{k=1}^{K}{\left[f\prime (k)-f(k)\right]}^{2}$$

where K is the dimension of the data set, *f*(*k*), *fn*(*k*) and f′(k) are the ideal input signal, noised input signal and processed signal respectively. Through different denoising methods, the SNR was improved and the MSE was decreased as shown in Table 1, which were calculated from 0.5 km to 1.5 km. The running time of every method was also summarized.

**Table 1:** The SNR, MSE and running time of different methods.

Method	R_SNR_input_	R_SNR_output_	E_MSE_input_	E_MSE_input_	Running Time (ms)
TLPF	15.4686	20.7557	0.555	0.1644	2.237
Triangular		21.6521		0.1425	3.233
Gaussian		23.5682		0.0985	3.512
SMF		18.7609		0. 2530	2.587
MF		20.9600		0.1569	2.106
WT		24.0133		0.0658	200.526
EMD		27.8862		0.0321	1612.534
PFFTF		27.6606		0.0335	2.855

From Figure 5 and Table 1, it can be seen that: (1) TLPF retained the fluctuation signal while it did not remove noise effectively; (2) SMF denoised the lidar signal, but removed the useful high frequency signal and lost some information; (3) MF, triangular filter and Gaussian filter retained the fluctuation signal information to some extent, however their denoising performance is not good enough; (4) running time of EMD and WT was far longer than that of the others, so EMD and WT are not reasonable methods; (5) among these methods, PFFTF removed noise better and additionally it retained the fluctuation signal better, therefore, we can conclude that PFFTF performs much better than other methods in lidar signal processing.

## 3. Lidar System

**Figure 6:**
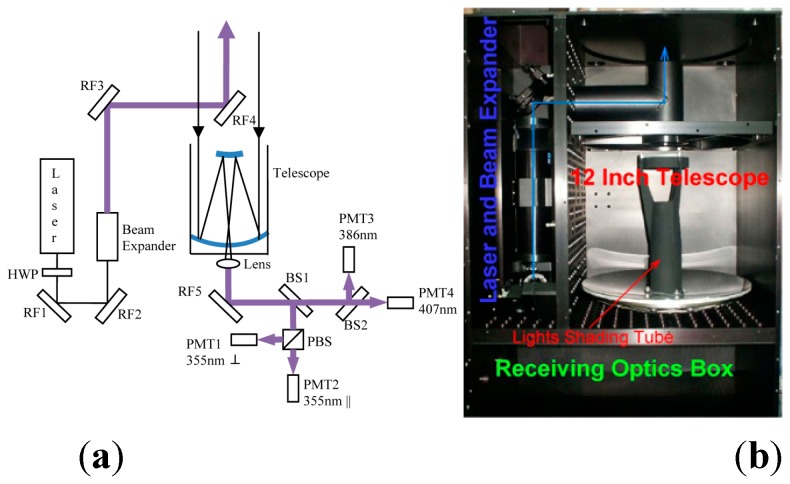
(**a**) Schematic diagram; (**b**) Inner structure.

A compact airborne Raman lidar system has been designed and assembled on a small aircraft. The design goal of the compact Raman lidar is to better study the planetary boundary layer aerosol and water vapor. The lidar needs to work during daytime. Under a strong daylight solar background, the laser power and telescope aperture are quite limited for an airborne system. Furthermore, in order to provide higher horizontally resolved measurements from aircraft, the averaging time of each lidar profile should be as short as possible. That means system requires the lidar signal processing to be fast and effective. A simplified instrument schematic diagram and inner structure of the compact Roman lidar system are shown in Figure 6.

In order to provide the essential stability, the transmitter, the telescope and the receiver optics are all mounted on the same optical bench to maintain the optical alignment in a vibration environment. A list of the main system parameters is given in Table 2.

**Table 2:** System parameters of compact airborne Raman lidar.

**Transmitter**			
Laser	Nd:YAG laser (Bigsky CFR400 GRM)		
Wavelength	354.7 nm		
Pulse energy	50 mJ		
Pulse width	7 ns		
Pulse Repetition Frequency (PRF)	30 Hz		
Beam divergence	1.8 mrad		
Beam expander	5X		
**Receiver**			
Telescope aperture	12 inch		
Field of View	1 mard		
Receiving Channels	4		
Polarization	Horizontal & Vertical		
Detector	PMT (Hamamatsu H5873)		
Filter center wavelength	354.7 nm	386.7 nm	407.5 nm
Filter Bandwidth (FWHM)	0.3 nm	0.3 nm	0.3 nm
Data Acquisition System	12-bit A/D (GAGE)		
Sampling rate	200 MSPS		

## 4. Experiments

**Figure 7:**
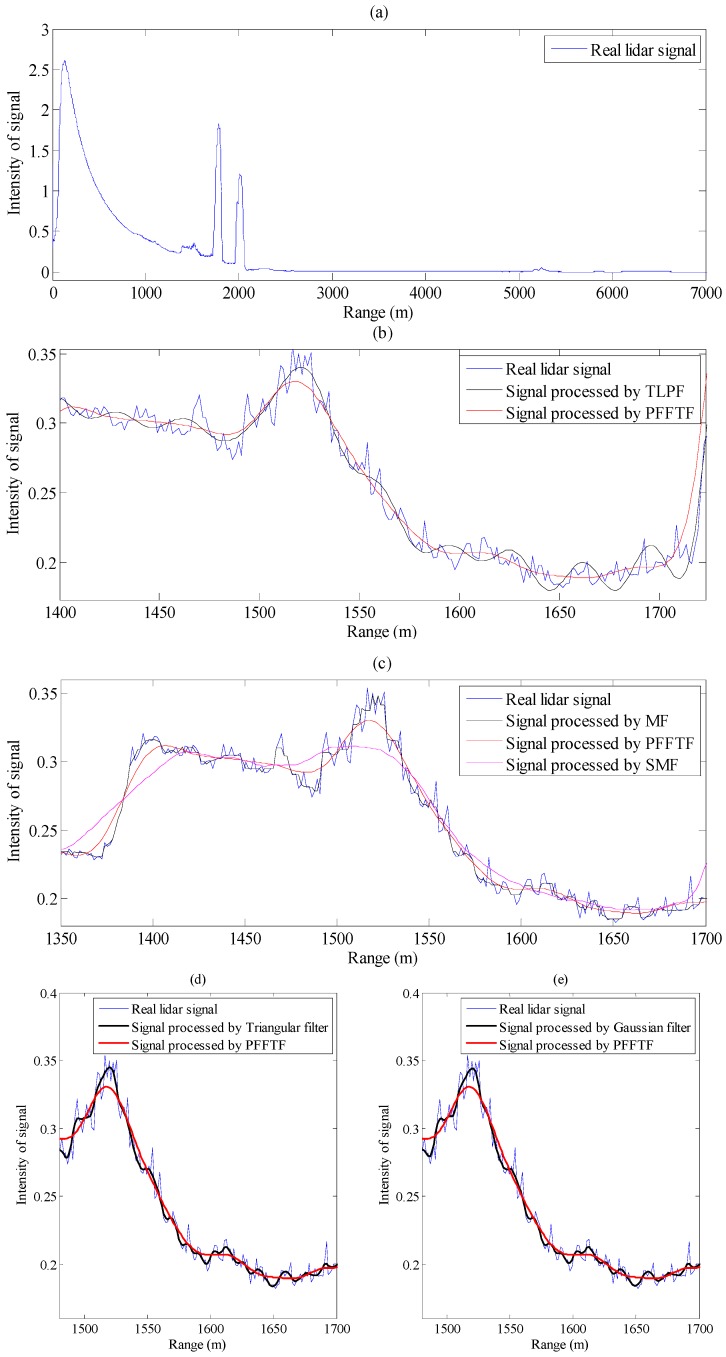
Processing of real lidar signal. (**a**) A whole lidar signal profile from the CARL; (**b**) Comparison between TLPF and PFFTF; (**c**) Comparison among PFFTF, MF and SMF; (**d**) Comparison between triangular filter and PFFTF; (**e**) Comparison between Gaussian filter and PFFTF.

The Compact Airborne Raman Lidar (CARL) was deployed on the Wyoming King Air during KAPEE project [10]. A single signal profile from the elastic channel of CARL was extracted for PFFTF demonstration and result of different methods was shown in Figure 7. The f_S_ is 100 MHz, f_C1_ is 10 Hz, f_C2_ is 4.36 MHz, cut off frequency of TLPF, triangular and Gaussian filter is 4.36 MHz, p is 2 for MF, m is 15 for SMF.

From Figure 7, it can be seen that there are several cloud layers in the lidar return profile. TLPF did not remove noise effectively between 1.4 km and 1.7 km. SMF distorted the cloud signals between 1.5 km and 1.55 km. For MF, triangular filer and Gaussian filter, the cloud signals were kept to some extent, but data was not denoised enough. PFFTF decreased the noise effectively while keeping the cloud signals well enough.

## 5. Conclusions

A low-pass parabolic fast Fourier transform filter was reported for lidar signal processing. It is evolved from an ideal low pass Fourier transform filter. Following minimum MSE criterion, relations between pass frequency & stop frequency of PFFTF and sampling frequency were studied by simulative analysis. Empirical formulas for optimal parameters of PFFTF were given through data fitting and the reasonability of formulas was verified. Comparisons of denoising effects by application of PFFTF, TLPF, triangular filter, Gaussian filter, SMF and MF for both simulated and real lidar signals were presented. Simulated computations show that PFFTF improved SNR best and had the minimum mean square error. Results for real lidar signal processing also proved the obvious advantages of PFFTF. To summarize, PFFTF is a dependable and effective method for the lidar signal denoising.

## References

[1] Ansmann W., Riebesell M., Weitkamp C. (1990). Measurement of Atmospheric Aerosol Extinction Profiles with a Raman Lidar. Opt. Lett..

[2] Wu S., Liu Z., Liu B. (2006). Enhancement of Lidar Backscatters Signal-to-Noise Ratio Using Empirical Mode Decomposition Method. Opt. Commun..

[3] Liu B., Wang Z. (2013). Improved Calibration Method for Depolarization Lidar Measurement. Opt. Express.

[4] Tian P., Cao X., Liang J., Zhang L., Yi N., Wang L., Cheng X. (2014). Improved Empirical Mode Decomposition Based Denoising Method for Lidar Signals. Opt. Commun..

[5] Liu B., Wu D., Fan A., Wang B., Yuan L., Bo G., Zhou J. (2011). Development of a Mobile Raman—Mie Lidar System for All Time Water Vapor and Aerosol Detection. J. Quant. Spectrosc. Radiat. Trans..

[6] Huang B.N.E., Shen Z., Long S.R., Wu M.C., Shih H.H. (1998). The Empirical Mode Decomposition and the Hilbert Spectrum for Nonlinear and Non-Stationary Time Series Analysis. Proc. R. Soc. Lond. A.

[7] Fang H., Huang D. (2004). Noise Reduction in Lidar Signal Based on Discrete Wavelet Transform. Opt. Commun..

[8] Kedzierski M., Fryskowska A. (2014). Terrestrial and Aerial Laser Scanning Data Integration Using Wavelet Analysis for the Purpose of 3D Building Modeling. Sensors.

[9] Whiteman D.N., Melfi S.H., Ferrare R.A. (1992). Raman Lidar System for the Measurement of Water Vapor and Aerosols in the Earth’s Atmosphere. Appl. Opt..

[10] Liu B., Wang Z., Cai Y., Wechsler P., Kuestner W., Burkhart M., Welch W. (2014). Compact Airborne Raman Lidar for Profiling Aerosol, Water Vapor and Clouds. Opt. Express.

[11] Liu Z., Zhang N., Wang R., Zhu J. (2007). Doppler Wind Lidar Data Acquisition System and Data Analysis by Empirical Mode Decomposition Method. Opt. Eng..

[12] Hook J. (2014). Smoothing Non-Smooth Systems with Low-Pass Filters. Phys. D. Nonlinear Phenom..

[13] Whiteman D.N. (2003). Examination of the Traditional Raman Lidar Technique. I. Evaluating the Temperature-Dependent Lidar Equations. Appl. Opt..

[14] Ansmann A., Wandinger U., Riebesell M., Weitkamp C., Michaelis W. (1992). Independent Measurement of the Extinction and Backscatter Profiles in Cirrus Clouds by Using a Combined Raman Elastic-Backscatter Lidar. Appl. Opt..

